# Solving inherited white matter disorder etiologies in the neurology clinic: Challenges and lessons learned using next-generation sequencing

**DOI:** 10.3389/fneur.2023.1148377

**Published:** 2023-04-03

**Authors:** Stefanie Perrier, Kether Guerrero, Luan T. Tran, Mackenzie A. Michell-Robinson, Geneviève Legault, Bernard Brais, Michel Sylvain, James Dorman, Michelle Demos, Wolfgang Köhler, Tomi Pastinen, Isabelle Thiffault, Geneviève Bernard

**Affiliations:** ^1^Department of Neurology and Neurosurgery, McGill University, Montreal, QC, Canada; ^2^Child Health and Human Development Program, Research Institute of the McGill University Health Centre, Montreal, QC, Canada; ^3^Department of Pediatrics, McGill University, Montreal, QC, Canada; ^4^Montreal Neurological Institute, McGill University, Montreal, QC, Canada; ^5^Department of Human Genetics, McGill University, Montreal, QC, Canada; ^6^Division of Pediatric Neurology, Centre Mère-Enfant Soleil du CHU de Québec - Université Laval, Québec City, QC, Canada; ^7^John H. Stroger Jr. Hospital of Cook County, Chicago, IL, United States; ^8^Department of Neurological Sciences, Rush Medical College, Chicago, IL, United States; ^9^Division of Neurology, Department of Pediatrics, University of British Columbia, BC Children's Hospital, Vancouver, BC, Canada; ^10^Leukodystrophy Center, University of Leipzig Medical Center, Leipzig, Germany; ^11^Genomic Medicine Center, Children's Mercy Hospital, Kansas City, MO, United States; ^12^University of Missouri Kansas City School of Medicine, Kansas City, MO, United States; ^13^Department of Pathology and Laboratory Medicine, Children's Mercy Hospital, Kansas City, MO, United States; ^14^Department of Specialized Medicine, Division of Medical Genetics, McGill University Health Center, Montreal, QC, Canada

**Keywords:** leukodystrophy, hypomyelination, next generation sequencing (NGS), genetic diagnosis, medical genetics, pediatric neurology, white matter disorders, neurogenetics

## Abstract

**Introduction:**

Rare neurodevelopmental disorders, including inherited white matter disorders or leukodystrophies, often present a diagnostic challenge on a genetic level given the large number of causal genes associated with a range of disease subtypes. This study aims to demonstrate the challenges and lessons learned in the genetic investigations of leukodystrophies through presentation of a series of cases solved using exome or genome sequencing.

**Methods:**

Each of the six patients had a leukodystrophy associated with hypomyelination or delayed myelination on MRI, and inconclusive clinical diagnostic genetic testing results. We performed next generation sequencing (case-based exome or genome sequencing) to further investigate the genetic cause of disease.

**Results:**

Following different lines of investigation, molecular diagnoses were obtained for each case, with patients harboring pathogenic variants in a range of genes including *TMEM106B, GJA1, AGA, POLR3A*, and *TUBB4A*. We describe the lessons learned in reaching the genetic diagnosis, including the importance of (a) utilizing proper multi-gene panels in clinical testing, (b) assessing the reliability of biochemical assays in supporting diagnoses, and (c) understanding the limitations of exome sequencing methods in regard to CNV detection and region coverage in GC-rich areas.

**Discussion:**

This study illustrates the importance of applying a collaborative diagnostic approach by combining detailed phenotyping data and metabolic results from the clinical environment with advanced next generation sequencing analysis techniques from the research environment to increase the diagnostic yield in patients with genetically unresolved leukodystrophies.

## 1. Introduction

Although genetic sequencing technologies have drastically evolved in recent years, identification and interpretation of rare variants associated with phenotypically similar but genetically heterogeneous diseases remains a challenge. Rare inherited white matter disorders, or leukodystrophies, can be especially difficult to genetically diagnose, given the growing number of causal genes associated with different disease subtypes (1, 2). Clinical presentation can be similar between patients, involving typically progressive neurological manifestations such as cerebellar, pyramidal, and extrapyramidal features, with or without cognitive involvement (3, 4). Upon MRI investigations to identify white matter abnormalities, leukodystrophies can be categorized based on imaging characteristics as hypomyelinating or as other pathologies, such as demyelinating leukodystrophies (5–7). In young children, it is important to differentiate hypomyelination from delayed myelination by repeating brain MRI as both have distinctive lists of differential diagnoses (6). Neuroimaging patterns and recognition of disease-specific MRI features can further aid in narrowing the underlying genetic cause of disease (5). On a clinical basis, diagnostic procedures typically combine brain MRI, genetic sequencing (e.g., multi-gene panels or exome sequencing) and metabolic investigations (e.g., enzyme deficiencies or CSF metabolite levels) to confirm or exclude diagnoses (3).

Since the rise of next generation sequencing (NGS) in the research environment, leukodystrophy diagnostic rates have seen significant increases, both in report of variants within known disease-associated genes, and in the discovery of novel disease-causing genes (8–12). Despite the increase in diagnostic rates, there still remain patients who are genetically unresolved following clinical and/or research investigations, which may result from limitations within the technology itself, challenges in variant identification, or evaluation of variant pathogenicity. Given that leukodystrophies can be associated with multi-systemic features, challenges may be faced when navigating differential genetic diagnoses, especially when presented with variants of unknown significance in multiple genes. Furthermore, when resolving the genetic basis of a leukodystrophy, it is essential to consider the patient's entire picture, including clinical presentation, disease progression, neuroimaging features, and metabolic test results.

This study presents an overview of several lessons learned in the diagnosis of patients with genetically unresolved white matter disorders. Each patient presented with hypomyelination or delayed myelination on MRI and was investigated genetically on a research basis by our laboratory after clinical testing remained inconclusive. We utilized NGS to determine the genetic cause of disease, noting the below challenges that were faced. The clinical and MRI features are described below with the corresponding genetic investigations, along with a discussion of the lessons learned on the path to resolving genetic diagnoses.

## 2. Methods

### 2.1. Protocol approvals, registrations, and patient consents

This research was approved by the Montreal Children's Hospital and McGill University Health Center Research Ethics Boards (11-105-PED; 2019-4972) and the Children's Mercy Institutional Review Board (study # 11120514). Informed consent was obtained from all participants and/or their parents/legal guardians.

### 2.2. Genetic sequencing and data interpretation

NGS data were either obtained from a clinical laboratory for further analysis or completed on a research basis using genomic DNA extracted from whole blood, fibroblasts, or saliva according to standard protocols. Exome sequencing (ES) and genome sequencing (GS) were performed on a case-by-case basis as described previously (13, 14). Potential disease-causing variants were identified and evaluated based on the *American College of Medical Genetics Standards and Guidelines for the Interpretation of Sequence Variants* (15). All variants were validated by Sanger sequencing or confirmed by clinical genetic testing. Medical records and brain MRIs were reviewed for each patient.

### 2.3. Data availability

Data supporting this study's findings are available upon reasonable request. Raw data from participants (i.e., raw genetic data and MRI data) are not publicly available to protect patient privacy. Pathogenic variants have been deposited in the National Center for Biotechnology Information (NCBI) ClinVar repository (ncbi.nlm.nih.gov/clinvar/) under the accession numbers: SCV002820973 - SCV002820979.

## 3. Results and discussion

For each of the patients described below, a genetic diagnosis was resolved following ES or GS completed on a research basis. We noted several challenges and lessons learned throughout our investigations, which are presented below and summarized in Table 1. Summaries of clinical and MRI features are described, along with genetic investigations and discussion of each lesson with insight on applications to future studies. Additionally, Table 2 describes the advantages and disadvantages of each sequencing modality utilized in this study (16–18).

**Table 1:** Summary of solved cases and lessons learned on the path to genetic diagnosis.

**Patient**	**Pathogenic variants**	**Diagnostic challenge**	**Lesson learned**
1	*TMEM106B* (NM_018374.3): c.754G>A; p.D252N	Causal gene absent on initial clinical gene panel despite publication one year prior	Importance of ensuring clinical panels include recently reported genes
2	*GJA1* (NM_000165.5): c.413G>A; p.G138N	Causal gene absent on initial clinical panel despite despite association with adulthood leukodystrophy and publication over a decade prior	Importance of including phenotypically compatible genes in initial clinical panel screening
3	*AGA* (NM_000027.4): c.319C>T; p.R107^*^ c.1018G>T; p.E340^*^	Reliability of biochemical assay support in confirming genetic diagnoses	If contradicting variant interpretation and biochemical results, consider reliability and usefulness of repeat testing
4 and 5 (siblings)	*POLR3A* (NM_007055.4): c.3013C>T, p.R1005C Deletion exons 6-8 (chr10:78020680-78023050del, GRCh38)	Limitations of exome sequencing in detecting multi-exon deletions	With identification of a single variant in a phenotypically compatible AR gene, consider secondary analysis *via* genome sequencing to ensure complete coverage for detection of all possible variants
6	*TUBB4A* (NM_006087.3): c.5G>A; p.R2Q	Limitations of exome sequencing in providing uniform coverage of all exons	If exome sequencing was completed several years prior, consider repeat exome sequencing using advanced platform for increased accuracy in variant detection

**Table 2:** Description of the advantages and disadvantages of using the three main discussed sequencing techniques: multi-gene panels, exome sequencing, and genome sequencing (16–18).

	**Multi-gene panels**	**Exome sequencing**	**Genome sequencing**
Sequenced region	Specific genes of interest associated with similar phenotypic presentations	Exons and intron-exon boundaries (~1% of the genome)	Entire genomic region including exons and introns (~90% of the genome)
Pros	• Higher read depth of desired regions • Curated set of genes associated with specific phenotype • Higher read coverage allows detection of somatic/mosaic variants • Mitochondrial genome can be targeted • Maximizes clinical sensitivity by avoiding detection of variant(s) in genes not related to reason of referral or lacking clinical validity, and avoiding non-coding region interpretation • Sanger sequencing and other technologies are commonly used to supplement gene panels, but impractical for exome/genome • Allow optimization of probe design or assay to overcome clinically significant regions affected by pseudogenes/segmental duplication or low complexity/repetitive elements • Typically clinically available • On a limited set of genes, the cost per base to achieve appropriate coverage is reduced	• High read depth of coding regions compared to genome sequencing (~130 × vs. 40 × ) • Generally high frequency of pathogenic variants in exons/intron-exon boundaries allows for efficient variant detection • Effective for conditions with heterogeneous phenotypes for which it may be difficult to choose a panel • Able to identify variants in novel genes not yet associated with disease • Supplementation with copy number backbone allows detection of copy number or structural variation with an overall sensitivity ~90%, including single exon/intragenic events • Higher read coverage allows detection of somatic/mosaic variants • Mitochondrial genome can be targeted • Allows for phenotype-driven (HPO) “virtual panel” analysis, an effective method to analyze exome data with high sensitivity • Reanalysis can be ordered • Lower cost per base than genome sequencing	• More even coverage of the entire genome • Allows for detection of pathogenic variants in non-coding regions (deep intronic, promotors, non-translated regions) • Increased capacity to detect copy number or structural variation as well as complex structural rearrangement (i.e., balanced/unbalanced translocation, inversion, insertion) and some repeat expansions • Able to identify variants in novel genes not yet associated with disease • Mitochondrial genome can be analyzed without targeted steps • Allows for phenotype-driven (HPO) “virtual panel” analysis • Reanalysis can be ordered • Faster workflow/turnaround time: does not require additional probes, reagents, or target enrichment steps in sequencing preparation pipeline
Cons	• Phenotypic variability of disease may present difficulty in choosing gene list • Causal gene may not be included in gene panel (limited guidance on gene inclusion/exclusion leads to highly variable gene content between clinical laboratories) • Less capability for copy number or structural variation detection • No capability for identifying variants in novel genes • Panel requires additional reagents (probes) and some additional steps (hybridization/enrichment) • Requires careful gene curation, test development and clinical validation each time panel content is modified, unless the panel is “virtually” extracted from a full exome sequencing	• Depth of coverage not uniform (some exonic regions may have low read coverage, resulting in the pathogenic variant being missed) • Unable to detect pathogenic variants in deep non-coding regions • Clinical information at the time of testing influences guidance on gene inclusion and reporting considerations for diagnostic gene (requires professional expertise) • Difficult to identify sequence and copy number or structural variation in regions with variable read depth i.e., GC-rich, repetitive regions or affected by pseudogenes due to read alignment • Requires additional reagents (probes) and some additional steps (hybridization/ enrichment) • Overall analytical sensitivity and specificity affected by enrichment bias, inadequate depth of coverage, or the accuracy of read alignment	• Lower depth of sequencing reads limits the detection of somatic/mosaic variants • Higher number of variants detected may reduce the analytical sensitivity and specificity • Higher cost per base • Large data storage requirement

### 3.1. Lesson I: Inclusion of phenotypically compatible genes in clinical gene-panel testing

The following two patients demonstrate the importance of utilizing updated and broad leukodystrophy-associated targeted gene panels in the context of clinical genetic testing. Furthermore, this applies to both patients with early-onset (Patient 1) and adult-onset (Patient 2) diseases.

#### 3.1.1. Patient 1: Clinical & MRI summary

Patient 1, a female, was born at term following a normal pregnancy, and presented shortly after birth with nystagmus, episodes of rapid tremor of the hands, and tremor of the mandibula while feeding. She had mildly reduced axial tone, and increased tone in all four limbs. Through infancy to early childhood, she continued to have mild to moderate axial hypotonia and spastic quadriparesis, with brisk deep tendon reflexes. She also had dysmetria, bilateral sensorineural hearing loss, and dysphagia. At age 3 years, she began to demonstrate additional neurological features, including dysarthria, mild sialorrhea, dysdiadochokinesis, and a slightly unstable gait. She had ophthalmic abnormalities involving strabismus and abnormal pursuits and saccades (hyper/hypometric). Despite showing an initial neurological deterioration, she later stabilized and then started improving, with developmental progress, resolution of sensorineural hearing loss, and amelioration of her dysphagia.

MRI at age 3 weeks revealed abnormal myelination, with myelin deposition being insufficient for age. At the time, due to her young age, it was not possible to determine if she had hypomyelination or myelination delay. There was also involvement of the corticospinal tracts in the c-spine, pyramids, and pons, as well as T2-hyperintensity of the cerebellar white matter. MRI at age 7 months revealed progression of the white matter signal changes, concerning both the deep and subcortical white matter and corpus callosum, and involvement of the middle cerebellar peduncles. An abnormal lactate peak was detected in the white matter on MR spectroscopy. Additionally, available sequences of the orbits demonstrated normal size but abnormal signal intensity of the intraorbital segments of the optic nerves characterized by T2 weighted imaging hyperintensity and increased signal on DWI, not confirmed in the ADC map.

#### 3.1.2. Patient 1: Genetic investigations

Following the initial abnormal MRI findings, genetic investigations for Patient 1 began with multi-gene panel sequencing on a clinical basis. After screening a panel of 163 known leukodystrophy and leukoencephalopathy genes, no conclusive variants were identified. On a research basis, ES was performed, leading to the identification of a *de novo* pathogenic variant in the gene *TMEM106B* (NM_018374.3): c.754G>A (p.D252N). This variant has been reported in six unrelated individuals with a hypomyelinating leukodystrophy as a single-nucleotide hotspot associated with this disease (19–21). In retrospect, this gene was not included in the clinical panel, and therefore the pathogenic variant was not detected during the first genetic investigation. The length of time between the first publication of the gene as disease-causing and the completion of the clinical multi-gene panel sequencing was approximately one year.

#### 3.1.3. Patient 2: Clinical & MRI summary

Patient 2, whose MRI was previously published in a Neuroimage case report (22), presented in adulthood with slowly progressive gait disturbances, falls, and issues with memory, starting at age 35. As a child, he was known to have stomach malrotation and syndactyly, the latter of which was surgically corrected. Despite minor motor problems since childhood, he reported normal activities of daily life, and remained fully ambulatory. He had slight dysmetria and mild gait ataxia. He was also noted to have mild facial dysmorphisms, dental abnormalities, and severe myopia.

MRI in adulthood revealed a pattern of diffuse hypomyelination, with involvement of the posterior limb of the internal capsule, pons, and cerebellar peduncles. T2-weighted hypointensities were also noted in both the Rolandic cortex and the dentate nucleus. The corpus callosum was thin and there was mild vermian atrophy (22).

Regarding significant family history, his mother also experienced slowly progressive neurodegeneration in adulthood, involving pyramidal signs and gait ataxia, worsening over several years. She experienced dysphagia, speech and cognitive difficulties, and epileptic seizures. She died in her early 80s after experiencing recurrent aspiration pneumonias.

Brain MRI of the patient's mother at age 80 showed diffuse white matter abnormalities, with involvement of the posterior limbs of the internal capsule and the pons (corticospinal tracts and medial lemniscus), along with significant atrophy of the posterior white matter. She also had a thin corpus callosum, cerebellar vermis atrophy, and ventriculomegaly (22).

#### 3.1.4. Patient 2: Genetic investigations

Following identification of white matter abnormalities on MRI of Patient 2, a gene panel including 122 leukodystrophy or leukoencephalopathy genes was completed on a clinical basis. Results were inconclusive, and the patient was referred to our research study for further investigations. ES was completed on DNA from the proband, revealing a heterozygous pathogenic missense variant in *GJA1* (NM_000165.5): c.413G>A; p.G138D. This variant was previously reported as pathogenic in association with autosomal dominant Oculodentodigital dysplasia (ODDD) syndrome (23). Parental DNA was unavailable for segregation analysis, but given the similar presentation of the mother, autosomal dominant inheritance was presumed. Notably, on the initial clinical gene panel, the *GJA1* gene (published over a decade prior) was not included, and therefore not identified on the first investigation.

#### 3.1.5. Inclusion of novel and compatible genes on clinical panels

Gene panels which target a specific set of genes known to be associated with disease phenotypes are often used as a first-line diagnostic tool in the clinical setting. While the use of gene panels may provide an effective means for identifying pathogenic variants in known genes, patients with pathogenic variants outside of those remain genetically undiagnosed, often leading to a long diagnostic odyssey. Advantages of using multi-gene panels compared to ES or GS have been studied in the past, and there remain benefits and limitations for each of these diagnostic techniques (Table 2) (24–28). While multi-gene panel sequencing can be a powerful molecular diagnostic tool, without inclusion of up-to-date causal genes, they remain limited in effectiveness for investigations of rare diseases. Regarding Patient 1, the *TMEM106B* gene was published as associated with hypomyelination nearly a year prior to the initial clinical screening (19). Contrarily, Patient 2 had a pathogenic variant in *GJA1*, published as associated with ODDD syndrome in the early 2000s (29). As neurological features are only seen in a portion of patients with ODDD, it is likely to be an often-unrecognized form of adulthood leukodystrophy. Thus, it remains possible it was excluded from the large leukodystrophy-associated gene panel due to the leukodystrophy features in ODDD remaining overlooked, as the phenotypic bias in diagnosis may lean toward the other associated features present on clinical evaluation.

Both above cases had to transition from clinical to research-based studies to be resolved, thus increasing the time to diagnosis, which could have been minimized had the initial panel screening contained the causal genes. Furthermore, these cases demonstrate the importance of maintaining clinical gene panels to be both updated with newly published genes and expansive enough to include likely causative genes. In the field of medical genetics, discovery of novel disease-associated genes advances at a rapid pace, with unique genetic causes of leukodystrophies continuously reported, which in parallel should be included in gene panels. It is also imperative that clinicians ordering clinical gene panels are knowledgeable regarding whether the proper genes are included when there is a high index of suspicion for a phenotypically distinct condition.

### 3.2. Lesson II: Reliability of biochemical assay support in confirming genetic diagnoses

Often, variants identified on ES may be classified as being of unknown significance if they have not been previously linked to a disease, or if functional evidence is lacking. For some implicated genes, clinical biochemical assay results may be used as biomarkers to support variant pathogenicity. The following case presents a scenario in which likely pathogenic variants were identified in a causal gene; however, this finding was initially unsupported by biochemical testing results. On repeat biochemical testing, results were verified and the genetic diagnosis was confirmed. Thus, this case demonstrates the importance of considering the validity of biochemical testing results when evaluating the likelihood of variant pathogenicity, especially when contradicting evidence is provided in the case of likely pathogenic variants.

#### 3.2.1. Patient 3: Clinical & MRI summary

Patient 3, a female, presented to the clinic with a wobbly gait at age 4 years and was found to have diffuse abnormal myelination on MRI, associated with a pattern of hypomyelination. MRI at age 5 was significant for diffuse hypomyelination/delayed myelination, where slight progression of myelination of the peripheral subcortical white matter was seen on T1-weighted imaging, with the degree of myelination on T2-weighted imaging remaining stable. Upon review of the latest MRI at age 8, there was also mild thinning of the corpus callosum, mild cerebellar vermis atrophy, and a mild increase in VR spaces, with T2-hypointensity of the pulvinar.

Clinically, she had a history of global developmental delay from age 6 months, and throughout childhood she continued to have mild developmental delay, without focal abnormalities. She was known for ophthalmic issues, including right eye esotropia and left optic nerve pallor. She had mild difficulties with tandem gait. Nerve conduction studies were normal. Through to age 8, she did not experience signs of regression, and only continued to have mild developmental challenges. She also had persistent mildly low platelets levels, which appeared to normalize at age 7. Initial urine oligosaccharides testing revealed a faint abnormal band, which was further investigated clinically *via* HPLC analysis, and reported to be unremarkable for the known lysosomal storage disorders (i.e., alpha mannosidosis, alpha fucidosis, sialidosis, galactosialidosis, GM1 gangliosidosis, GM2 gangliosidosis type Sandhoff, GSD11 Pompe disease infantile). On biochemical testing of CSF, amino acids and neurotransmitter metabolites were unremarkable.

#### 3.2.2. Patient 3: Genetic investigations

ES was performed using patient genomic DNA, and upon initial data analysis, two compound heterozygous nonsense variants were identified in the gene *AGA* (NM_000027.4): c.319C>T; p.R107^*^, and c.1018G>T; p.E340^*^. *AGA* encodes for the enzyme aspartylglucosaminidase, and pathogenic variants which impair its function are known to cause aspartylglucosaminuria (OMIM: 208400), an autosomal recessive neurodegenerative lysosomal storage disease. The first *AGA* nonsense variant (p.R107^*^) results in a truncated protein product lacking 240 amino acids (aa) from the 347-aa length wildtype protein. Although this specific variant has not been reported in published cases, similar truncating variants are known to be associated with AGA loss-of-function and cause disease (30, 31). The second nonsense variant (p.E340^*^) results in a truncated protein lacking only 7-aa compared to the wildtype protein. While this variant has also not been reported previously, the nearby p.E334^*^ variant has been studied functionally to cause a reduction in the production of an active enzyme (30). Based on ACMG criteria (15), both variants were predicted to be likely pathogenic. Each variant was validated using Sanger sequencing, however, parental DNA was unavailable to confirm segregation.

Regarding confirmatory biochemical testing, diagnosis of aspartylglucosaminuria is supported by screening urine oligosaccharides, as aspartylglucosamine accumulates in the urine of affected individuals (32). In this patient, initial urine oligosaccharides analysis revealed an abnormal band, however, further HPLC analysis did not detect a pattern associated with disease. This result contradicted with the predicted pathogenic variant interpretation as both variants were thought to be associated with impaired enzyme function. Therefore, it was decided to repeat clinical urine oligosaccharides screening to verify the results, and on the second screen, an abnormal pattern associated with aspartylglucosaminuria was detected, providing support for the diagnosis. Additional tests were performed, including a sialic acid assay which showed mild elevation of total sialic acid, thought to result from abnormal accumulation of an oligosaccharide species associated with aspartylglucosaminuria. Finally, enzymatic testing demonstrated low aspartylglucosaminidase activity, further confirming the diagnosis of aspartylglucosaminuria.

#### 3.2.3. Importance of biochemical assay results in supporting genetic diagnoses

Biochemical and functional assays are an important diagnostic component for many genetic disorders as metabolic results often provide clues to a diagnosis, such as in lysosomal storage disorders. In the presented case, initial urine oligosaccharides screening results did not support the most likely candidate gene, and therefore difficulties were met in resolving the diagnosis. Variants were predicted to be pathogenic, however, we were unable to confirm their inheritance on trans alleles. In addition, biochemical testing provided conflicting evidence, thus causing uncertainty in either the genetic or biochemical results. The diagnosis was only confirmed after repeated biochemical screening for urine oligosaccharides, which also resulted in a prolonged diagnostic period. Therefore, this case demonstrates the importance of maintaining a degree of trust in variant interpretation guidelines when evaluating pathogenicity, and that repeat biochemical screening may be necessary should results contradict the genetic diagnosis. This case also stresses the importance of remaining cautious when presented with conflicting diagnostic evidence and approaching the reassessment of either genetic or biochemical results with a high level of clinical reasoning. Contrary to what was seen in our case, in which molecular genetic investigations allowed for the diagnosis of a metabolic disease where the biochemical genetic tests were initially normal, it is possible that genetic variants initially thought to be likely pathogenic can be reclassified as benign based on biochemical investigations. For example, in considering pathogenic variants in the *ABCD1* gene which cause adrenoleukodystrophy, the detection of true pathogenic variants is complicated by the fact that several non-functional pseudogenes exist on different chromosomes, which may result in false-positive variant detection (33). In this case, it is crucial to confirm the pathogenicity of the variants with very long chain fatty acids. In conclusion, special attention should be given to cases with conflicting genetic and biochemical evidence, and next steps for confirming or rejecting diagnoses should be considered only after critical evaluation of all provided evidence.

### 3.3. Lesson III: Understanding limitations of exome sequencing in variant detection

While the use of ES has demonstrated high success rates in resolving genetic causes of many rare diseases, it is well-known that this technology is associated with several limitations in its capacity to detect all disease-causing variants (34). The following three cases illustrate the shortcomings of ES in variant detection, including the limited ability to detect CNVs (Patients 4 and 5) and variants in GC-rich regions (Patient 6).

#### 3.3.1. Patients 4 and 5: Clinical & MRI summary

Patients 4 and 5 were siblings who each experienced increasing cognitive and behavioral concerns in early adulthood and were found to have hypomyelination on MRI. Patient 4, a female, had behavioral problems since childhood, with intellectual and learning disabilities. She had severe myopia, as well as growth and pubertal abnormalities, experiencing menarche at age 14 but requiring hormonal treatment for irregular menstruations. In early adulthood, she was diagnosed with bipolar disorder, and further MRI investigations revealed white matter abnormalities, leading to a diagnosis of leukodystrophy. In adulthood, she experienced further neurological deterioration, exhibiting dysarthria, intention tremor, gait ataxia, as well as dystonia. She also had saccadic pursuits, with limitations in upgaze. She further experienced dysphagia and required a gastrostomy. Delayed dentition was noted in childhood, and in adulthood it was reported her teeth were becoming loose. MRI at age 30 years was significant for hypomyelination with a pattern compatible with POLR3-related leukodystrophy. There was also moderate diffuse cerebral and cerebellar atrophy. Interestingly, bone abnormalities were noted in the skull, described as diffuse thickening of the calvarium.

Patient 5, a male, had onset of behavioral issues and personality changes in his late 20s. His development was reported as normal, having graduated high school after attending special education classes. He was reported to have puberty and growth abnormalities, receiving growth hormone treatment in early adolescence. He had severe myopia but reported no dental abnormalities through childhood. In early adulthood, he began to experience cognitive decline, with severely impaired intellectual function, while behavioral difficulties, specifically irritability, persisted. Family noted episodes of pseudobulbar affect. He was reported to have a high-pitched voice, but no dysarthria. He had hypotonia and mild gait ataxia. Brain MRI in adulthood revealed hypomyelination with a pattern compatible with POLR3-related leukodystrophy. Similar to his sister, he also had thickening of the calvarium.

#### 3.3.2. Patients 4 and 5: Genetic investigations

ES was first completed on both siblings, and one pathogenic missense variant was discovered in *POLR3A* (NM_007055.4): c.3013C>T; p.R1005C. This variant was previously reported in a compound heterozygous state in patients with POLR3-related leukodystrophy (35, 36). As POLR3-related leukodystrophy is an autosomal recessive condition and only one variant was identified, data reanalysis was performed to attempt to identify an alternate genetic cause, however, no strong candidates were found. GS was then completed, identifying a maternally inherited *POLR3A* deletion of approximately 3 kb on 10q22.3, including exons 6-8 [(chr10:78020680-78023050)x1, GRCh38], thereby resolving the genetic diagnosis as POLR3-related leukodystrophy. Notably, a similar deletion of *POLR3A* exons 6-8 (NG_029648.1; NC_000010.11: g.78020702_78023071del; c.646–687_c.1185+844del; p.E216_K395del) has been associated with spastic ataxia when in compound heterozygous state with the *POLR3A* c.1909+22G>A variant (37). Additionally, large deletions in the RNA polymerase III subunit gene *POLR3B*, which forms the catalytic core of the enzyme along with POLR3A, are associated with a similar phenotype of POLR3-related leukodystrophy (38). This diagnosis aligns with the clinical features seen in both patients including the typical MRI pattern, severe myopia, and growth and endocrine abnormalities, as well as the delayed dentition in Patient 4.

#### 3.3.3. Advantages of genome sequencing in CNV detection

ES is known for several limitations in variant detection, inherent to its technological design. Moreover, this includes the inability to detect certain types of variants, including CNVs like the large deletion described in the above cases. This limitation is a direct result of the lack of sequencing depth uniformity in ES, as the enriched exonic regions interspaced by non-coding intronic regions are not evenly sequenced. Therefore, deletions are difficult to detect through conventional ES methods, and GS offers a more effective means to resolve the deleted region. While ES generally captures a higher sequencing depth of the targeted exonic regions, GS offers a more uniform coverage of the genome (39). Indeed, as it provides more even and unbiased coverage of all exonic regions, GS delivers more accurate variant detection, especially in the recognition of large deletions (40). The described cases offer a practical example of the limitations of ES in detecting CNVs, demonstrating the importance of pursuing additional investigations when a single pathogenic variant is identified in a gene associated with a phenotypically compatible autosomal recessive disease. The *POLR3A* gene could have been investigated in more detail on a research basis through long-range PCR, primer walking, or quantitative PCR to resolve the genetic diagnosis. Considering the limited ability to perform such experiments in the clinical setting, GS is a better approach, and in this case, the benefits of GS were clearly demonstrated as it provided an efficient means to detect the deletion. In conclusion, these cases demonstrate a lesson in harnessing knowledge of the limitations associated with variant detection *via* ES when investigating genetic diseases.

#### 3.3.4. Patient 6: Clinical & MRI summary

Patient 6, a male, was born at 40 weeks and 6 days following an induced vaginal delivery, and was treated for hyperbilirubinemia with phototherapy for 2 days. Shortly after birth, he presented with nystagmus, and further experienced severe developmental delay and failure to thrive. He demonstrated neurological features including severe axial and appendicular hypotonia, moderate spasticity, and generalized dystonia. He was severely delayed in motor development, unable to gain normal head control or the ability to sit independently or walk. He experienced sialorrhea and dysphagia, requiring a gastrostomy at age 3. He had epilepsy from early childhood, which was well-controlled by age 11. He also experienced asymptomatic subluxation of both hips. Additionally, he had bilateral myopia and strabismus, with bilateral optic atrophy and restriction of extraocular movements. At age 13, his condition was relatively stable, although he had slight motor deterioration, losing the ability to pick up objects with his hands.

Brain MRIs demonstrated diffuse hypomyelination with relative preservation of the cerebellum and brainstem. On follow-up MRIs, cerebral and cerebellar atrophy were noted.

#### 3.3.5. Patient 6: Genetic investigations

Patient 6 was first investigated using ES on a research basis, however, no strong candidates were identified despite data reanalysis. Five years after the initial sequencing, we opted to repeat ES using an updated platform in an attempt to identify variants which may have been previously undetected. This resulted in identification of a *de novo* pathogenic variant in *TUBB4A* (NM_006087.3): c.5G>A; p.R2Q, resolving the diagnosis. This variant was reported in two individuals having hypomyelination with atrophy of the basal ganglia and cerebellum (H-ABC) (41, 42). Although *TUBB4A* variants are mainly associated with H-ABC, isolated hypomyelination has also been described (42–45), aligning with Patient 6's MRI pattern, which did not display basal ganglia involvement.

Upon review of the initial ES data, the genomic region containing the *TUBB4A* variant was only covered by five sequencing reads, only one of which carried the variant. Therefore, due to suboptimal coverage and read depth, the variant was not detected on the first investigation. Indeed, this variant was located near the beginning of the gene's first exon, a region generally known to contain an increased GC content (46) and consequently associated with reduced coverage on ES.

#### 3.3.6. Coverage bias in exome sequencing and impact on variant detection

The case of Patient 6 is a direct example of the limitations of ES in detecting variants in areas commonly subject to coverage bias. It is well-known that ES is associated with non-uniform coverage in specific regions, including those that are GC-rich. Uneven coverage of these regions may result from technological challenges in either library or cluster amplification, the sequencing step itself, or the alignment of sequencing data (47–49). Updates to sequencing platforms aim to mitigate this through advanced methods, which have led to more consistent coverage in recent years. Therefore, when evaluating the best course of action for persistently unsolved cases sequenced several years prior, it is important to consider the benefits of re-sequencing using updated platforms. Through this case, the value of re-sequencing was certainly demonstrated as a diagnosis was easily resolved on the second round of sequencing, with the causal variant being located in an area which was not initially covered on first round sequencing, and thereby not detected. Furthermore, it is important to remain knowledgeable of the efficacy and utility of sequencing technologies used in genetic analyses, and whether updated technologies for repeat sequencing could be valuable for subsequent investigations.

## 4. Conclusion

In this study, we illustrate several lessons learned when investigating genetic diagnoses in a subset of patients with previously genetically unresolved leukodystrophies following negative clinical investigations. The encountered challenges represent common lessons and pitfalls that clinicians and researchers may face when navigating pathogenic variant identification and interpretation in genetic diagnostics. The first two cases highlight the importance of utilizing appropriate gene panels in first line clinical investigations, ensuring that they contain both recently published and phenotypically compatible genes. The next case demonstrates the importance of using clinical reasoning when evaluating biochemical results that conflict with probable genetic diagnoses. Finally, the last three cases illustrate the technical limitations associated with variant detection *via* ES, which were resolved using repeat sequencing to identify previously undetected variants. When investigating genetically unresolved cases, remaining knowledgeable of the challenges associated with variant identification can provide insight on the most beneficial and effective course of action. However, it is important to note that although technological limitations may impede detection of pathogenic variants, cases could remain genetically unresolved due to other factors, such as challenges in accurate variant pathogenicity interpretation due to gaps in knowledge base, or simply because the causal gene has not yet been associated with a disease. It is imperative that unsolved cases are regularly re-evaluated as the field advances in both sequencing technologies, analysis techniques, and reports of novel genes. Notably, protocols for the genetic diagnosis of both pediatric and adult-onset leukodystrophies can aid clinicians in evaluating the most optimal approach to sequencing. Several molecular diagnostic workflows and step-by-step approaches based on large cohorts and expert consensus recommendations have been described in the literature, which can provide guidance in the clinical setting (3, 50–54). Furthermore, in a clinical context, resolving genetic diagnoses of rare inherited diseases is especially important for understanding of the disease course and prognosis, as well as tailoring supportive care, and further advising through genetic counseling.

## Data availability statement

The data presented in the study are deposited in the National Center for Biotechnology Information (NCBI) ClinVar repository (ncbi.nlm.nih.gov/clinvar/) under the accession numbers: SCV002820973 - SCV002820979.

## Ethics statement

The studies involving human participants were reviewed and approved by the Montreal Children's Hospital and McGill University Health Center Research Ethics Boards (11-105-PED; 2019-4972) and the Children's Mercy Institutional Review Board (study # 11120514). Written informed consent to participate in this study was provided by the participants and/or their legal guardians/next of kin.

## Author contributions

SP, IT, and GB designed the study, analyzed and interpreted the data, and drafted the manuscript. KG, LT, MM-R, GL, BB, MS, JD, MD, WK, and TP contributed to the acquisition and interpretation of the clinical and genetic data and revised the manuscript for intellectual content. GB and IT oversaw the entire study. GB, IT, and TP secured funding for the study. All authors approved the final version of the manuscript.
